# Predictive models for endoscopic disease activity in patients with ulcerative colitis: Practical machine learning-based modeling and interpretation

**DOI:** 10.3389/fmed.2022.1043412

**Published:** 2022-12-21

**Authors:** Xiaojun Li, Lamei Yan, Xuehong Wang, Chunhui Ouyang, Chunlian Wang, Jun Chao, Jie Zhang, Guanghui Lian

**Affiliations:** ^1^Department of Gastroenterology, The Second Xiangya Hospital of Central South University, Research Center of Digestive Disease, Central South University, Changsha, China; ^2^Department of Gastroenterology, The First Affiliated Hospital of Shaoyang College, Shaoyang, Hunan, China; ^3^Hunan Aicortech Intelligent Research Institute Co., Changsha, Hunan, China; ^4^Department of Gastroenterology, Xiangya Hospital of Central South University, Changsha, Hunan, China

**Keywords:** ulcerative colitis, machine learning, SHAP, predictive models, endoscopic disease activity, mayo endoscopic score, ulcerative colitis endoscopic index of severity

## Abstract

**Background:**

Endoscopic disease activity monitoring is important for the long-term management of patients with ulcerative colitis (UC), there is currently no widely accepted non-invasive method that can effectively predict endoscopic disease activity. We aimed to develop and validate machine learning (ML) models for predicting it, which are desired to reduce the frequency of endoscopic examinations and related costs.

**Methods:**

The patients with a diagnosis of UC in two hospitals from January 2016 to January 2021 were enrolled in this study. Thirty nine clinical and laboratory variables were collected. All patients were divided into four groups based on MES or UCEIS scores. Logistic regression (LR) and four ML algorithms were applied to construct the prediction models. The performance of models was evaluated in terms of accuracy, sensitivity, precision, F1 score, and area under the receiver-operating characteristic curve (AUC). Then Shapley additive explanations (SHAP) was applied to determine the importance of the selected variables and interpret the ML models.

**Results:**

A total of 420 patients were entered into the study. Twenty four variables showed statistical differences among the groups. After synthetic minority oversampling technique (SMOTE) oversampling and RFE variables selection, the random forests (RF) model with 23 variables in MES and the extreme gradient boosting (XGBoost) model with 21 variables in USEIS, had the greatest discriminatory ability (AUC = 0.8192 in MES and 0.8006 in UCEIS in the test set). The results obtained from SHAP showed that albumin, rectal bleeding, and CRP/ALB contributed the most to the overall model. In addition, the above three variables had a more balanced contribution to each classification under the MES than the UCEIS according to the SHAP values.

**Conclusion:**

This proof-of-concept study demonstrated that the ML model could serve as an effective non-invasive approach to predicting endoscopic disease activity for patients with UC. RF and XGBoost, which were first introduced into data-based endoscopic disease activity prediction, are suitable for the present prediction modeling.

## 1 Introduction

Ulcerative colitis (UC) is an idiopathic inflammatory disorder affecting the colon and rectum, with an increasing incidence worldwide (1, 2). To date, the etiology and pathogenesis of UC are not well clarified, and this disease remains incurable (3). Current therapy of UC focuses on the induction and maintenance of endoscopic remission, which is associated with clinical remission, fewer hospitalizations, and abdominal surgeries (1, 4, 5). Due to the characteristic of repeated recurrence, most patients require long-term or even life-long treatment. During such processes, frequent monitoring of UC disease activity is crucially important, as it can guide dose and regimen adjustments to reduce the risk of recurrence, which in turn improves the long-term survival rate and quality of life in patients with UC (6, 7). As an essential assessment of UC disease activity, colonoscopy can help clinicians determine the status of intestinal mucosal lesions directly, which is crucial to evaluating disease extent and severity. However, colonoscopy as an invasive examination is often an unpleasant experience for patients, and these patients have to bear the economic burden and risks of serious complications at the same time. In addition, the epidemic of COVID-19 has made it more difficult for patients to undergo colonoscopy (8). Therefore, a convenient and accurate method to evaluate endoscopic disease activity is needed.

In UC, the inflammatory disease activity scoring systems are preferably established by endoscopy (9, 10). Non-endoscopic disease activity indices, such as the Seo Index and simple clinical colitis activity index, can also quantify the severity of the disease and predict prognosis clinically (10, 11). However, non-endoscopic disease activity indices fail to correlate well with endoscopically proven intestinal inflammation (10, 12). Moreover, some clinical scales in disease activity scoring systems include a degree of subjectivity, so the results can be biased. Some biochemical markers, such as C-reactive protein (CRP), erythrocyte sedimentation rate (ESR), fecal calprotectin (FC), and fecal immunochemical test, have been proposed as indicators of the extent of UC. Although these indicators have the advantages of being non-invasive and repeatable, they still have limited sensitivity and specificity, and some of them have not been widely performed (10, 13–16). For example, FC, which has a good predictive ability of mucosal healing, is limited by the popularity of the kit and cannot be widely practiced under the influence of the COVID-19 epidemic. Many previous studies attempt to establish the predicting models by using symptomatic, laboratory, endoscopic, radiological, or pathological features, and most of them employed statistical methods such as univariate and multivariate analyses to search for the predictors (17–20). These models sometimes are relatively difficult to be broadly applied and optimized owing to high demands on the amount and quality of the data. Therefore, an efficient strategy needs to be developed and adopted to address the above problems.

In recent years, machine learning (ML) has emerged as a powerful tool in medicine, primarily owing to its discriminatory and decision-making capabilities. ML algorithms have the characteristics of continuously updating learning and capturing relationships among variables, which can be a good approach to solving the problems in UC disease activity prediction model building. Previous studies have demonstrated that ML models can provide better accuracy and discrimination for the diagnosis of inflammatory bowel disease (IBD), prediction of biologic treatment response in UC patients, and prognoses of patients with acute severe colitis (21–25). It creates opportunities for exploring the relationships among features and building highly efficient models. Automated image recognition using deep learning methods also has been applied in the endoscopic images and pathological images recognition of IBD (21, 26–28). Moreover, in search of new well-performing markers at the gene and microbiome level, the ML methods showed the greatest contribution in variables screening (29, 30). Based on the development of these techniques, introducing ML into the area of UC evaluation can provide a promising approach for researchers. Previous studies of ML for predicting gut disease severity have focused on patients with Crohn’s disease. Nevertheless, to the best of our knowledge, there have been no previous attempts to use ML algorithms based on clinical data and laboratory tests to predict endoscopic activity in patients with UC (25). The implementation of this ML predictive model can provide physicians and patients with useful information on endoscopic disease activity, which would be of great benefit to UC patients who require long-term management.

Herein, we performed a study on the endoscopic severity of inflammation for patients diagnosed with UC, and collected the clinical characteristics, laboratory data, and endoscopic results. Then, logistic regression (LR), random forests (RF), extreme gradient boosting (XGBoost), multilayer perceptron (MLP), and support vector machine (SVM) models were built to analyze and predict endoscopic severity. The proposed framework consists of three components. First, we performed the imbalanced treatment of the dataset using a synthetic minority oversampling technique (SMOTE) algorithm, then five models were built to predict endoscopic disease activities in UC. At the last, the best model was demonstrated through Shapley values. This study introduces ML to endoscopic disease activity prediction in UC for the first time. We aim to identify variables and establish a prediction model of UC endoscopic disease activity based on generally available clinical information. The model can also help monitor and guide medicating for UC, which may avoid frequent colonoscopy examinations.

## 2 Materials and methods

### 2.1 Study population

This cohort study included patients from the Department of Gastroenterology, Second Xiangya Hospital, and Department of Gastroenterology, Xiangya Hospital, Central South University. The case collection was conducted from January 2016 to January 2021. The inclusion criteria for this study were adult patients (age ≥ 18 years) with a confirmed diagnosis of UC. Patients with malignancy, chronic or severe underlying diseases were excluded. We assert that all procedures contributing to this work comply with the ethical standards of the relevant national and institutional committees on human experimentation and with the Helsinki Declaration of 1975, as revised in 2008. The research was approved by the Ethics Committee of the Second Xiangya Hospital of Central South University (NO. 20181230). The data are anonymous, and written informed consent for participation was therefore waived for this study following the national legislation and the institutional requirements.

### 2.2 Diagnostic criteria

Diagnosis of UC was based on the Consensus on Diagnosis and Treatment of Inflammatory Bowel Disease (2012, Guangzhou) (31). The endoscopic status of the UC patients was assessed according to the Mayo endoscopic score (MES) (32) and ulcerative colitis endoscopic index of severity (UCEIS) (33). All endoscopic examinations were performed by gastroenterologists who were experienced in IBD and optical diagnosis. The MES and UCEIS were obtained from endoscopy reports written by certified gastroenterologists. Here, MES 0 and UCEIS 0 were defined as endoscopic remission; MES 1 and UCEIS 1–3 were defined as mild disease activity; MES 1 and UCEIS 4–6 were defined as moderate disease activity; MES 3 and UCEIS 7–8 as severe disease activity. The score of stool frequency and rectal bleeding were assessed using the modified Mayo scoring system (34), that is, stool frequency: 0 = normal; 1 = 1–2 stools more than normal; 2 = 3–4 stools more than normal; 3 = 5 or more stools more than normal. Rectal bleeding: 0 = no blood seen; 1 = streaks of blood with stool less than half the time; 2 = obvious blood with stool most of the time; 3 = blood alone passed. Classification of patient disease location according to Montreal classification: E1 = Ulcerative proctitis, E2 = Left sided UC (distal UC), E3 = Extensive UC (pancolitis).

### 2.3 Data collection and analysis

According to expert advice and literature review, clinically relevant data of the participants were recorded including demographic data, clinical manifestation, laboratory examinations, medication history, and endoscopic findings.

Demographic data were as follows: age, gender, weight, family history, history of abdominal operations including appendectomy and other surgeries, history of alcohol, and smoking history. Clinical manifestations were as follows: body temperature, pulse rate, decrease of weight in recent 1 year, stool frequency, rectal bleeding, disease duration, and disease location. The score of stool frequency and rectal bleeding were assessed using the modified Mayo scoring system. Laboratory examinations were as follows: white blood cells, hemoglobin, platelets, neutrophils, lymphocyte, monocyte, eosinophilia, basophils, mean corpuscular volume, hematocrit, red cell distribution width, mean platelet volume, plateletcrit, albumin, ESR, CRP, CRP/albumin (CRP/ALB), serum calcium, urea, and fecal calprotectin. Medication history includes history of 5-aminosalicylate (5-ASA), hormone, azathioprine, thalidomide, anti-tumor necrosis factor (TNF), and other biologics. Endoscopic findings were assessed according to the MES and UCEIS.

A total of 420 UC patients were included in the analysis. Thirty-nine variables were first analyzed for their predicting power of endoscopic disease activity in UC patients. All patients were divided and assigned to four groups based on endoscopic disease activity score (remission, mild, moderate, or severe). All data were presented as means ± standard errors of the means (SEM), medians (quartile range), or proportions with corresponding percentages (*n*, %).

#### 2.3.1 Variable screening and data processing

**FIGURE 1:**
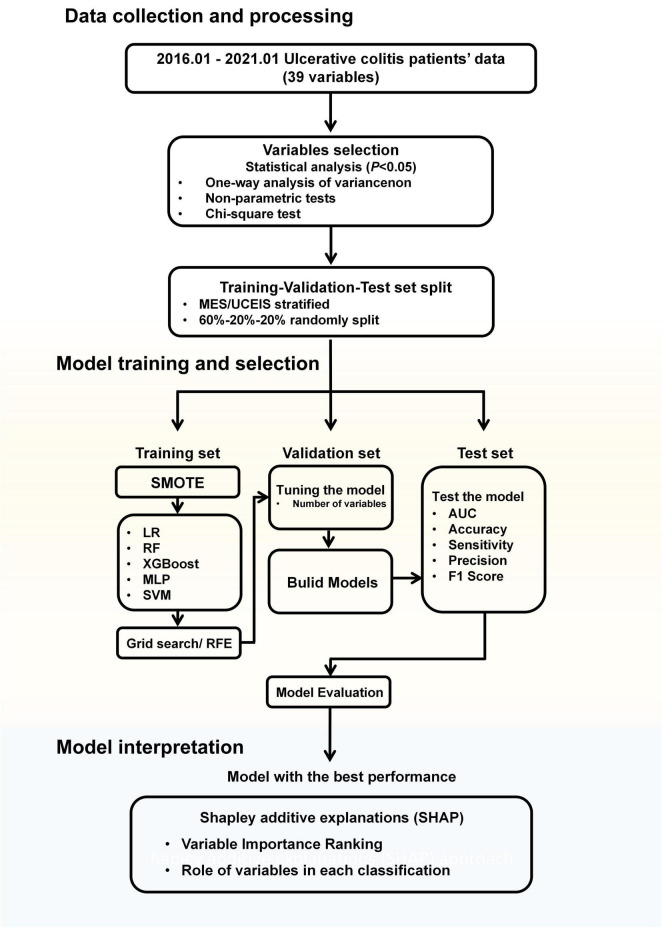
Workflow diagram. MES, mayo endoscopic subscore; UCEIS, ulcerative colitis endoscopic index of severity; SMOTE, synthetic minority oversampling technique; LR, logistic regression; RF, random forests; XGBoost, extreme gradient boost; MLP, multilayer perceptron; SVM, support vector machine; RFE, recursive feature elimination; AUC, area under the receiver-operating characteristic curve.

The work flow diagram of this research is shown in Figure 1. Variables were compared with each other among four groups. We used One-way analysis of variance (ANOVA) for data with normal distributions, non-parametric tests for data without normal distributions, and the Chi-square test was used to compare enumeration data. Statistical significance was expressed as a *P*-value with a significance level of 0.05. We carried out the variable selection to remove invalid variables containing irrelevant or redundant information.

Then all data were stratified into a training set, validation set, and test set randomly according to the MES or UCEIS level, with the distribution of 60% as a training set for model training, 20% as a validation set for the model tuning, and remaining 20% as the test set for model performance evaluation.

The importance of each variable was assessed using the recursive feature elimination (RFE) algorithm in the training set, with all variables being sorted according to their level of importance (35). After the variables had been sequentially reduced in the order of importance, the remaining variables were introduced into the corresponding ML algorithm.

During the model’s initialization, imbalanced datasets cause performance loss in the classification model. The models tend to predict the sample as the category with the majority of samples. To address the serious imbalance in the number of patients with different disease activities, we used the SMOTE in model training to tackle the data imbalance problem. SMOTE generates a synthetic instance by interpolating the *m* instances (for a given integer value m) of the minority class that lies close enough to each other to achieve the desired ratio between the majority and minority classes (36).

The performance of all models was measured with accuracy, sensitivity, precision (positive predictive value), F1 score (macro-weighted), and macro-area under the receiver-operating characteristic curve (AUC). The best performance is determined by maximizing the AUC. To translate the 4-class model scores to the metrics investigated, a one-vs.-all analysis of the scores was performed. By comparing the values of the models in the test dataset, we determined the model with the best predictive performance. The Delong test was used to compare the differences in the performance of the different models.

#### 2.3.2 Prediction model building

Predictive models were built using selected informative variables with the help of LR, RF, XGBoost, MLP, and SVM classification algorithm in Python. All models were trained in the training set, the optimal number of variables was adjusted in the validation set, and finally, the models’ performance was compared in the test set.

##### 2.3.2.1 Logistic regression

Logistic regression is one of the most common and widely applied methods used for the analysis. The algorithm of LR has been detailed elsewhere (37). Pre-screened variables were taken for further LR analyses. The regression coefficients of the predictive model were regarded as the weights for the respective variables, and the score for each patient was calculated. For each sample, the probabilities of each degree were calculated and the class with the highest probability was the classification result of this sample.

##### 2.3.2.2 Random forests

Random forests is an ensemble learning algorithm generating decision trees based on the training data. In training, models have been built using the full 60% of the training data, automatic tuning of hyperparameters (number of trees and maximum depth of the tree) was performed by using the grid search (scikit-learn GridSearchCV) (38). This tuning process was repeated for each possible combination of parameter values in the training set. These predictions were summarized to one outcome per participant by majority voting.

##### 2.3.2.3 Extreme gradient boosting

Extreme gradient boosting is also a kind of tree-based ML method. It uses multiple (hundreds of) classification and regression trees, which can learn non-linear relations among input variables and outcomes in a boosting ensemble manner, to capture and learn non-linear and complex relations accurately. It has been widely used in classification and regression tasks. One of the major advantages of using this algorithm is that XGBoost provides L1 and L2 regularization. L1 regularization handles sparsity, whereas L2 regularization reduces overfitting (39). Hyperparameters tuning was performed by using the grid search (the number of trees, learning rate, and maximum tree depth).

##### 2.3.2.4 Multilayer perceptron

Multilayer perceptron can have one or more non-linear hidden layers between the input and output layers. MLP can be utilized to construct effective classifier algorithms for distinguishing data that are not linearly separable (40). We trained the MLP model with one hidden layer, the best hyperparameters were determined using the grid search (number of nodes for hidden layer).

##### 2.3.2.5 Support vector machine

Support vector machine is a supervised learning method that constructs a hyperplane or set of hyperplanes in a high- or infinite-dimensional space used for classification. It does not build a model for each class, but only finds the discriminative hyperplane with the largest margin determined by the support vectors from the training data (41). Here, we used SVM on the training dataset to predict the disease activity.

The algorithms and the statistical analysis were implemented in Python 3.5.2 (Python Software Foundation, Wilmington, DE, USA). All automatic tuning of hyperparameters and the models were created using the scikit-learn package library (version 0.22.2) except the XGBoost model which was created by the XGBoost package library (version 1.1.1).

### 2.4 Model interpretation

The model with the highest AUC in the test set was regarded as the best model, which was included for further analysis. Although it is possible to visualize which variables have a greater impact on the model, it is hard to determine the relationship between the variables and results. Therefore, the Shapley additive explanations (SHAP) approach is applied to further model interpretation. SHAP is a method that allows for variable interpretation of non-linear black-box ML models (42). It is a game theory-based model explanation and is the only theoretically supported explanation currently. The mean absolute value of the SHAP values for each variable represents their average contribution to the overall model predictions, and it can clarify whether the influence of a variable is positive or negative. Compared to other methods that simply rank importance or decision direction, SHAP combines the influence of variable importance and trend characteristics of variables, to explain the variables in the model in a multidimensional way. SHAP values of the variables were calculated, and were further analyzed for clarifying the main role of each variable in the model prediction. SHAP values were computed and visualized with the SHAP Python package (version 0.29.1).

## 3 Results

### 3.1 Patient population and baseline characteristics

A total of 420 patients were entered into the study. According to the MES, patients were classified as MES remission group (*n* = 18), MES mild group (*n* = 57), MES moderate group (*n* = 183), and MES severe group (*n* = 162). According to the UCEIS, patients were classified as UCEIS remission group (*n* = 16), UCEIS mild group (*n* = 74), UCEIS moderate group (*n* = 282), and UCEIS severe group (*n* = 48). The mean age of enrolled patients was 45.1 ± 13.0 years, and 63.57% (267/420) of patients were male. Group analysis according to the different definitions of outcomes was performed. Thirty-nine variables from UC patients were evaluated by one-way ANOVA variance analysis, non-parametric tests, and Chi-square analysis. Twenty-four variables showed statistical differences among the four groups (*P* < 0.05). Fecal calprotectin was removed before modeling because the missing rate was > 50%. Finally, 23 variables were selected as candidate variables for further analysis (Table 1).

**TABLE 1:** Analysis of the clinical and laboratory variables in patients with ulcerative colitis.

Variables	MES					UCEIS				
	MES remission (*n* = 18)	MES mild (*n* = 57)	MES moderate (*n* = 183)	MES severe (*n* = 162)	*P*-value	UCEIS remission (*n* = 16)	UCEIS mild (*n* = 74)	UCEIS moderate (*n* = 282)	UCEIS severe (*n* = 48)	*P*-value
**Clinical variables**										
Age (year)	38.5 ± 15.0	44.3 ± 13.1	46.4 ± 12.6	44.6 ± 13.1	0.070	39.8 ± 15.5	45.9 ± 12.8	45.6 ± 12.9	42.5 ± 13.3	0.146
Gender (male, %)	14 (77.8%)	33 (57.9%)	124 (67.8%)	96 (59.3%)	0.169	13 (81.2%)	47 (63.5%)	176 (62.4%)	31 (64.6%)	0.505
Disease duration	27.0 (14.6, 42.6)	24.0 (12.0, 90.0)	24.0 (10.0, 60.0)	22.5 (5.8, 49.2)	0.245	29.5 (16.3, 46.3)	29.5 (11.8, 44.0)	22 (8.8, 50)	12 (1.3, 69)	0.031
Smoking history	0 (0%)	11 (19.3%)	40 (21.9%)	37 (22.8%)	0.152	0 (0%)	15 (20.3%)	66 (24.4%)	7 (14.6%)	0.092
Disease location					0.000					0.000
E1	12 (66.7%)	36 (63.2%)	69 (37.7%)	27 (16.7%)		10 (62.5%)	45 (60.8%)	84 (29.8%)	5 (10.4%)	
E2	6 (33.3%)	21 (32.8%)	114 (62.3%)	135 (83.3%)		6 (37.5%)	29 (39.2%)	198 (70.2%)	43 (89.6%)	
Stool frequency (0: 1: 2: 3)	13:5:0:0 72.2%: 27.8%: 0%: 0%	14:30:8:5 24.6%: 52.6%: 14.0%: 8.8%	6:63:46:68 3.3%: 34.4%: 25.1%: 37.2%	2:15:47:98 1.2%: 9.3%: 29.0%: 60.5%	0.000	12:4:0:0 75%: 25%: 0%: 0%	14:32:13:15 18.9%: 43.2%: 17.6%: 20.3%	9:73:75:125 3.2%: 25.9%: 26.6%: 44.3%	0:4:13:31 0%: 8.3%: 27.1%: 64.6%	0.000
Rectal bleeding (0: 1: 2: 3)	18:0:0:0 100%: 0%: 0%: 0%	14:21:15:7 24.6%: 36.8%: 26.3%: 12.3%	20:39:98:26 10.9%: 21.3%: 53.6%: 14.2%	5:30:95:32 3.1%: 18.5%: 58.6%: 19.8%	0.000	16:0:0:0 100%: 0%: 0%: 0%	17:24:23:10 23.0%: 32.4%: 31.1%: 13.5%	22:60:159:41 7.8%: 21.3%: 56.4%: 14.5%	2:6:26:14 4.2%: 12.5%: 54.2%: 29.2%	0.000
Pulse rate	69.3 ± 6.6	79.0 ± 9.9	80.3 ± 11.0	85.9 ± 13.1	0.000	68.8 ± 5.8	78.8 ± 9.8	82.2 ± 12.0	88.1 ± 13.8	0.000
Temperature (^°^C)	36.6 ± 0.3	36.5 ± 0.2	36.6 ± 0.3	36.6 ± 0.4	0.310	36.6 ± 0.3	36.6 ± 0.2	36.6 ± 0.4	36.6 ± 0.3	0.700
Weight (kg)	56.6 ± 9.4	57.8 ± 9.2	57.4 ± 11.0	54.0 ± 9.7	0.011	58.0 ± 8.9	59.1 ± 11.4	55.3 ± 10.1	55.8 ± 9.8	0.030
Decrease of weight	0.0 (0.0, 0.0)	0.0 (0.0, 2.5)	0.0 (0.0, 4.0)	3.5 (0.0, 6.0)	0.000	0.0 (0.0, 0.0)	0.0 (0.0, 2.0)	1.0 (0.0, 5.0)	5.0 (0.5, 8.5)	0.000
**Medications**										
5-ASA	17 (94.4%)	46 (80.7%)	152 (83.1%)	127 (78.4%)	0.343	15 (93.8%)	65 (87.8%)	229 (81.2%)	33 (68.8%)	0.033
Hormone	7 (38.9%)	3 (5.3%)	72 (39.3%)	121 (74.7%)	0.000	7 (43.8%)	15 (20.3%)	142 (50.4%)	39 (81.2%)	0.000
Immunomodulator	6 (33.3%)	0 (0%)	1 (0.5%)	2 (1.2%)	0.000	6 (37.5%)	0 (0%)	2 (0.7%)	1 (2.1%)	0.000
**Laboratory variables**										
White blood cells	6.11 ± 2.10	6.55 ± 1.96	7.97 ± 2.91	9.20 ± 3.83	0.000	6.01 ± 1.98	6.89 ± 2.20	8.44 ± 3.38	9.28 ± 3.91	0.000
Hemoglobin	139.22 ± 16.90	129.60 ± 22.13	122.64 ± 24.25	104.65 ± 24.61	0.000	141.44 ± 12.68	129.66 ± 20.99	114.64 ± 26.32	106.31 ± 24.21	0.000
Platelets	215.78 ± 69.76	234.93 ± 90.30	284.05 ± 112.56	353.88 ± 131.27	0.000	212.13 ± 71.78	245.15 ± 95.84	315.01 ± 128.92	337.88 ± 113.82	0.000
Neutrophils	3.99 ± 1.90	4.15 ± 1.59	5.56 ± 2.74	6.75 ± 3.28	0.000	3.90 ± 1.78	4.48 ± 1.94	5.99 ± 2.98	7.04 ± 3.53	0.000
Lymphocyte	1.94 ± 0.82	1.73 ± 0.70	1.67 ± 0.72	1.49 ± 0.61	0.006	1.97 ± 0.87	1.70 ± 0.63	1.61 ± 0.70	1.42 ± 0.55	0.024
Monocyte	0.3 (0.2, 0.4)	0.4 (0.3, 0.6)	0.4 (0.3, 0.7)	0.4 (0.6, 0.9)	0.000	0.3 (0.2, 0.4)	0.4 (0.3, 0.6)	0.5 (0.4, 0.7)	0.6 (0.4, 0.9)	0.036
Plateletcrit	0.23 (0.22, 0.27)	0.21 (0.18, 0.28)	0.26 (0.20, 0.31)	0.31 (0.25, 0.39)	0.000	0.23 (0.22, 0.27)	0.22 (0.19, 0.27)	0.28 (0.21, 0.35)	0.31 (0.23, 0.37)	0.000
Hematocrit	43.28 ± 4.07	39.52 ± 6.23	37.56 ± 6.77	32.60 ± 6.64	0.000	43.86 ± 2.99	39.19 ± 6.66	35.48 ± 7.09	32.93 ± 6.69	0.000
MCV	86.6 (86.3, 91.1)	91.0 (86.1, 94.0)	88.8 (85.0, 93.1)	86.1 (80.3, 91.0)	0.005	86.6 (86.3, 91.6)	90.8 (86.3, 93.8)	87.3(82.6,91.6)	87.1 (82.7, 91.0)	0.059
MPV	10.85 ± 1.60	10.28 ± 2.03	9.96 ± 1.72	9.31 ± 1.53	0.000	10.92 ± 1.66	10.13 ± 2.01	9.67 ± 1.62	9.62 ± 1.83	0.009
Urea	4.8 (3.9, 5.7)	4.1 (3.3, 5.0)	4.3 (3.4, 5.4)	3.6 (2.6, 4.6)	0.025	4.7 (3.9, 5.6)	4.2 (3.5, 5.2)	4.0 (3.1, 5.2)	3.4 (2.3, 4.5)	0.159
Albumin	46.86 ± 2.19	40.58 ± 5.43	37.47 ± 6.05	30.76 ± 6.05	0.000	46.95 ± 2.30	40.93 ± 5.17	34.68 ± 6.70	29.88 ± 6.13	0.000
ESR	4.5 (2.8, 8.0)	12.0 (5.5, 23.5)	26.0 (11.0, 46.0)	43.0 (25.8, 66.3)	0.000	4.0 (2.3, 7.0)	12.0 (5.8, 24.5)	33.0 (16.0, 56.3)	42.0 (25.3, 67.3)	0.000
CRP	1.5 (0.2, 2.3)	3.0 (1.7, 5.0)	6.0 (3.0, 24.0)	31.5 (11.0, 77.3)	0.000	1.5 (0.2, 2.4)	3.0 (1.5, 6.3)	13.0 (4.0, 42.0)	24.0 (10.9, 78.3)	0.000
CRP/ALB	0.03 (0, 0.05)	0.06 (0.04, 0.13)	0.15 (0.07, 0.70)	1.05 (0.36, 2.86)	0.000	0.04 (0.00, 0.05)	0.08 (0.04, 0.19)	0.39 (0.10, 1.31)	0.83 (0.33, 3.14)	0.000
FC	28.4 ± 12.6	62.7 ± 32.5	302.1 ± 253.2	225.1 ± 153.0	0.005	28.4 ± 12.6	78.8 ± 44.5	246.9 ± 219.0	247.0 ± 165.3	0.030

Values are means ± standards errors (SEM), median (quartile range), or *n* (%). MES, mayo endoscopic subscore; UCEIS, ulcerative colitis endoscopic index of severity; 5-ASA, 5-aminosalicylic acid; MCV, mean corpuscular volume; ESR, erythrocyte sedimentation rate; MPV, mean platelet volume; CRP, C-reactive protein; ALB, albumin; FC, fecal calprotectin.

### 3.2 Variables selection and model construction

After data were stratified into a training, validation, and test set, the proportion of each level of MES or UCEIS is similar among the three sets. Moreover, patients were similar in age and gender distribution among the sets (Supplementary Figures 1, 2).

Supplementary Table 1 shows the ranking of the variables based on the permutation importance method in RFE algorithm in the training set. The results of permutation importance demonstrated that the top two variables were albumin and CRP/ALB in both MES and UCEIS, through the process of RFE variables selection, we determined the optimal variable numbers and AUC of each algorithm. The prediction of endoscopic disease activity was carried out with LR, RF, XGBoost, MLP, and SVM classifiers, the full results of hyperparameters automatic tuning can be found in Supplementary Table 2.

**FIGURE 2:**
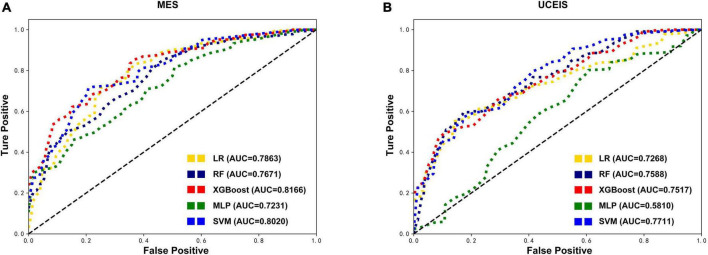
Comparison of the original test datasets-based models’ performance. Receiver operating characteristic curves showing the endoscopic disease activity predictive performance of five algorithms based on the mayo endoscopic subscore (MES) **(A)** and ulcerative colitis endoscopic index of severity (UCEIS) **(B)** in test datasets. LR, logistic regression; RF, random forests; XGBoost, extreme gradient boost; MLP, multilayer perceptron; SVM, support vector machine; AUC, area under the receiver-operating characteristic curve.

First, we built the model based on the original training set with all variables. According to the MES, the best predictive performance in the test set was observed in XGBoost (AUC = 0.8166), followed by SVM (AUC = 0.8020), LR (AUC = 0.7863), RF (AUC = 0.7671), and MLP (AUC = 0.7231). The XGBoost model outperformed the other algorithm-based models with the highest AUC, accuracy, sensitivity, precision, and F1 score (Table 2 and Figure 2A). And according to the UCEIS, the best-performing models ranking order are SVM (AUC = 0.7711), followed by RF (AUC = 0.7588), XGBoost (AUC = 0.7517), LR (AUC = 0.7268), and SVM (AUC = 0.5810). The SVM model had the highest AUC and accuracy. However, we observed the highest values of sensitivity (0.4473), precision (0.6194), and F1 score (0.4877) in the LR model (Table 2 and Figure 2B). In original datasets, comparing the performance of all models in MES and UCEIS classification groups revealed that, although the accuracy of the model with UCEIS was better than that of the model with MES, the AUC, sensitivity, precision, and F1 score of the model were higher in MES, which might be primarily due to the more unbalanced class of the original data in UCEIS.

**TABLE 2:** The performance of the models with all variables in the test set.

Algorithms	Group	Variables number	Accuracy	Sensitivity	Precision	F1 Score	AUC
LR	MES original	23	0.5882	0.4920	0.5384	0.5077	0.7863
	MES SMOTE	23	0.5357	0.5215	0.5323	0.5117	0.7956
	UCEIS original	23	0.6352	0.4473	0.6194	0.4877	0.7268
	UCEIS SMOTE	23	0.5556	0.5125	0.4890	0.4957	0.7518
RF	MES original	23	0.5349	0.3561	0.3578	0.3510	0.7671
	MES SMOTE	23	0.6046	0.6102	0.6554	0.6258	0.8192
	UCEIS original	23	0.6744	0.3157	0.3016	0.3008	0.7588
	UCEIS SMOTE	23	0.6279	0.4807	0.5177	0.4915	0.7851
XGBoost	MES original	23	0.6046	0.4957	0.6795	0.5269	0.8166
	MES SMOTE	23	0.5873	0.6332	0.5770	0.5722	0.8183
	UCEIS original	23	0.6627	0.3572	0.5344	0.3808	0.7517
	UCEIS SMOTE	23	0.6979	0.5317	0.5756	0.5363	0.7958
MLP	MES original	23	0.4235	0.3370	0.4948	0.3735	0.7231
	MES SMOTE	23	0.4000	0.3658	0.5310	0.3105	0.6876
	UCEIS original	23	0.6588	0.2701	0.2370	0.2500	0.5810
	UCEIS SMOTE	23	0.2941	0.3570	0.3286	0.2857	0.6824
SVM	MES original	23	0.5697	0.4062	0.4138	0.4048	0.8020
	MES SMOTE	23	0.5556	0.4759	0.4970	0.4585	0.8164
	UCEIS original	23	0.6744	0.3247	0.5436	0.3145	0.7711
	UCEIS SMOTE	23	0.6825	0.5036	0.4356	0.4445	0.7863

LR, logistic regression; RF, random forests; XGBoost, extreme gradient boost; MLP, multilayer perceptron; SVM, support vector machine; AUC, area under the receiver-operating characteristic curve; MES, mayo endoscopic subscore; UCEIS, ulcerative colitis endoscopic index of severity; SMOTE, synthetic minority oversampling technique.

**FIGURE 3:**
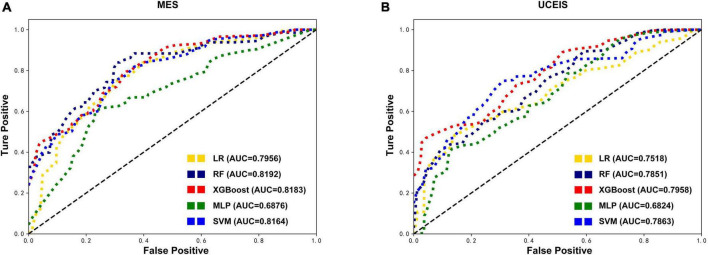
Comparison of the oversampling datasets-based models’ performance. After the synthetic minority oversampling technique (SMOTE) method, receiver operating characteristic curves show the endoscopic disease activity predictive performance of five algorithms based on the mayo endoscopic subscore (MES) **(A)** and ulcerative colitis endoscopic index of severity (UCEIS) **(B)** in test datasets. LR, logistic regression; RF, random forests; XGBoost, extreme gradient boost; MLP, multilayer perceptron; SVM, support vector machine; AUC, area under the receiver-operating characteristic curve.

Except for the MLP, all other models showed an increase in AUC after SMOTE oversampling, with the most notable being the RF model. The algorithms with SMOTE application outperformed the algorithms with original datasets in most models (*P* < 0.05). The RF model performed best with the highest AUC (0.8192) in MES-based datasets, and had the best accuracy (0.6046), precision (0.6554), and F1 score (0.6258). After the RF model, the XGBoost model ranked second in model performance (AUC = 0.8183), which had the best sensitivity (0.6332). The models based on SVM and LR algorithms slightly underperformed than RF and XGBoost models. Meanwhile, the model performance of MLP was worse than the original data in MES-based datasets instead. Although the sensitivity and precision of the MLP model have increased, the AUC still decreased significantly, this situation may be caused by the MLP not being able to simulate the data well after the noise amplification caused by SMOTE. In UCEIS-based datasets, the XGBoost model performed best with the highest AUC (0.7958), accuracy (0.6979), sensitivity (0.5317), precision (0.5756), and F1 score (0.5363), followed by SVM (AUC = 0.7863), RF (AUC = 0.7851), LR (AUC = 0.7518), and MLP (AUC = 0.6824) (Table 2 and Figure 3). With the above approach, we identified the training set after the SOMTE method as the base dataset for model building, and the MES-based data was modeled using the best-performing RF algorithm, while the UCEIS-based data was modeled using the XGBoost algorithm.

Then through the process of RFE feature selection and SOMTE, the optimal variable numbers and AUCs of each algorithm were determined (Table 3). The results revealed that the prediction model with the highest AUC (0.8508) in the validation set was the RF model based on the top 23 variables in MES. Moreover, the model also showed good performance in the test set (AUC = 0.8192). In UCEIS, the AUC of the XGBoost model (0.8140) with 21 variables was higher than that of the XGBoost model with 23 variables (0.7940) in the validation set. So, we choose the XGBoost model with 21 variables as best performed model in the USEIS dataset, and this model achieved an AUC of 0.8006 in the test set. Other model scores had a slight decrease after reduction, but the AUC increased instead, considering the improvement of model overfitting after reducing the variables. As described above, according to the model performance, we chose the RF model with 23 variables in the MES-SMOTE dataset the and XGboost model with 21 variables in UCEIS dataset as our final prediction model.

**TABLE 3:** The best performance of the models in the validation set and test set.

Algorithms	Set	Group	Variables number	Accuracy	Sensitivity	Precision	F1 score	AUC
RF	Validation	MES SMOTE	23	0.6046	0.4987	0.5402	0.5065	0.8508
		MES SMOTE	22	0.5813	0.4968	0.5350	0.5011	0.8390
	Test	MES SMOTE	23	0.6046	0.6102	0.6554	0.6258	0.8192
XGBoost	Validation	UCEIS SMOTE	23	0.6470	0.5846	0.4906	0.5229	0.7940
		UCEIS SMOTE	21	0.6350	0.5803	0.4832	0.5167	0.8140
	Test	UCEIS SMOTE	23	0.6979	0.5317	0.5756	0.5363	0.7958
		UCEIS SMOTE	21	0.6979	0.5195	0.6109	0.5277	0.8006

RF, random forests; XGBoost, extreme gradient boost; AUC, area under the receiver-operating characteristic curve; MES, mayo endoscopic subscore; UCEIS, ulcerative colitis endoscopic index of severity; SMOTE, synthetic minority oversampling technique.

### 3.3 Model interpretation

**FIGURE 4:**
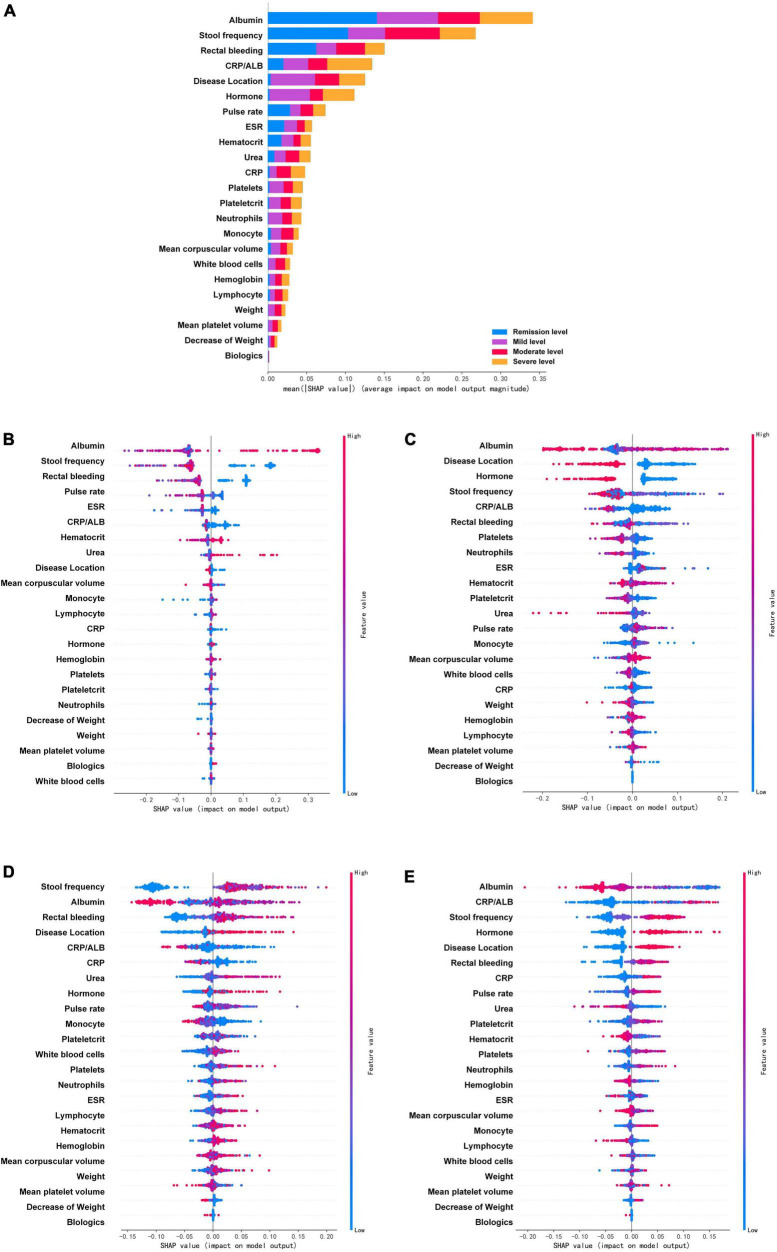
Feature importance ranking based on Shapley additive explanations (SHAP) values in mayo endoscopic subscore based RF model. **(A)** The contribution of each variable to the overall model. The different colors represent the contribution given by the variable under that classification. **(B–E)** Analysis of all variables’ SHAP values at each classification level, each point in the figure represents a sample. The variables are ranked according to the sum of the SHAP values for all patients in the remission level **(B)**, mild level **(C)**, moderate level **(D)**, and severe level **(E)**. Red indicates that the value of a variable is high, and blue indicates that the value of a variable is low. The x-axis indicates the effect of SHAP values on the model output. The larger the value of the x-axis, the greater the probability of this level. ESR, erythrocyte sedimentation rate; CRP, C-reactive protein; ALB, albumin.

**FIGURE 5:**
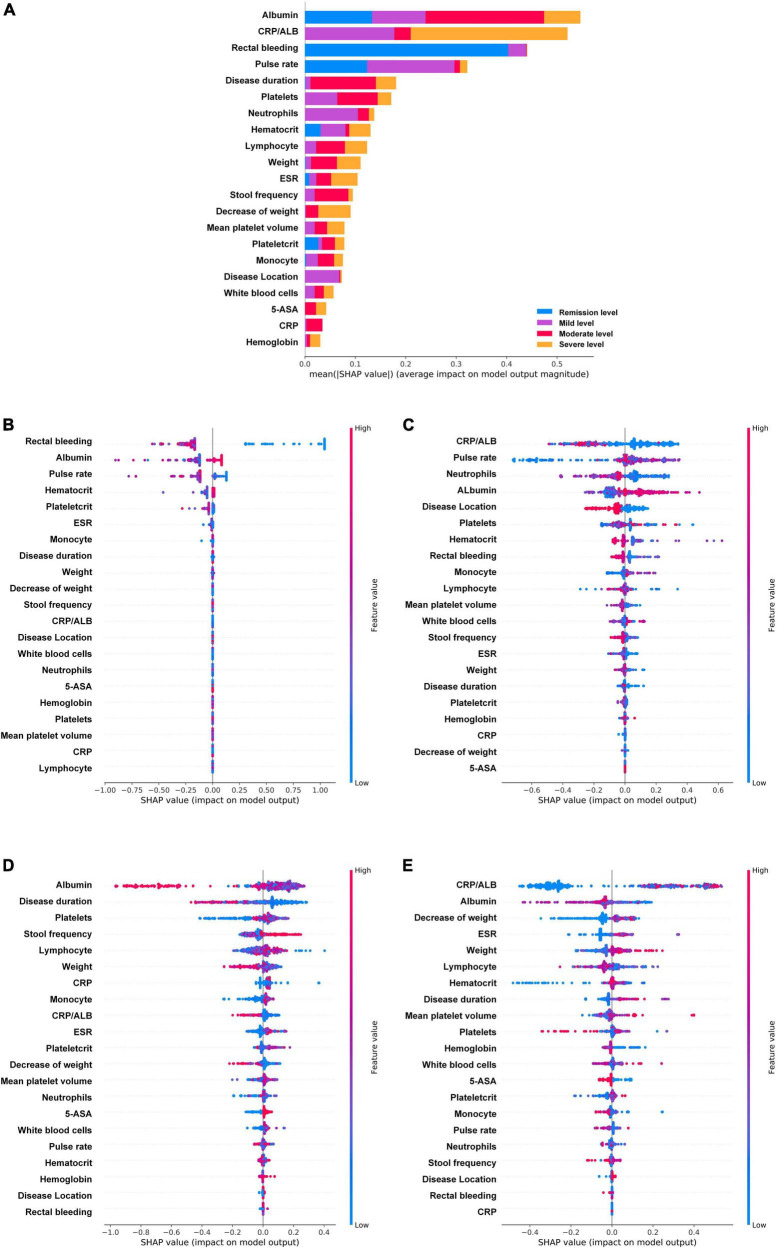
Feature importance ranking based on Shapley additive explanations (SHAP) values in ulcerative colitis endoscopic index of severity score based XGboost model. **(A)** The contribution of each variable to the overall model. The different colors represent the contribution given by the variable under that classification. **(B–E)** Analysis of all variables’ SHAP values at each classification level, each point in the figure represents a sample. The variables are ranked according to the sum of the SHAP values for all patients in the remission level **(B)**, mild level **(C)**, moderate level **(D)**, and severe level **(E)**. Red indicates that the value of a variable is high, and blue indicates that the value of a variable is low. The x-axis indicates the effect of SHAP values on the model output. The larger the value of the x-axis, the greater the probability of this level. ESR, erythrocyte sedimentation rate; CRP, C-reactive protein; ALB, albumin; 5-ASA, 5-aminosalicylic acid.

To further understand and get an overview on the importance of the variables, SHAP was implemented for global model interpretation. SHAP scores are feature importance scores based on Shapley values from game theory, and the SHAP value for the same variable may differ across patients. Figures 4, 5 showed the main contribution of each variable in the model prediction of endoscopic disease activity. The different colors represent the contribution given by the variable under that classification.

In the MES, the prediction model based on RF after SMOTE was analyzed by SHAP (Figure 4). The four variables that were found to contribute most to the overall model were albumin, stool frequency, rectal bleeding, and CRP/ALB. These four variables contributed significantly to all level classifications. Albumin and stool frequency contributed similarly to the four classifications. Rectal bleeding primarily contributed to the discrimination of the remission level and CRP/ALB primarily contributed to that of the severe level. Most of the variables contributed to the classification of the four levels, except for neutrophils, decrease of weight, weight, mean platelet volume, white blood cells, and biologics, which contributed almost nothing to the discrimination of remission level. In particular, biologics did not play a significant role in all classifications, but the model efficacy decreased significantly after the deletion of this variable during the model tuning, which may be due to its intrinsic correlation with other variables. The importance of the variables under each classification was further analyzed. Figures 4B–E showed the analysis of all variables’ SHAP values at each classification level, each point in the figure represents a sample. The horizontal coordinate represents the Shapley corresponding to each feature of each sample. A positive value indicates that the prediction probability of this classification would be improved. In the remission level prediction, albumin, stool frequency, rectal bleeding, and pulse rate had stronger effects on model prediction according to SHAP. The result showed that negative rectal bleeding, negative stool frequency, albumin, and pulse rate in the normal range were associated with an increased likelihood of remission level, which is also consistent with clinical experience. In addition, biologics and white blood cells made a negligible contribution to the prediction of the remission level. In the mild level prediction, the top four variables contributing to the model were albumin, disease location, hormone, and stool frequency in order. Albumin in the normal range, disease location in the rectum, no previous use of hormones, and negative stool frequency suggested an increased likelihood of the mild level. While, in the order of predictors’ importance at the moderate level, the top four variables in terms of contribution were stool frequency, albumin, rectal bleeding, and disease location. Increased stool frequency, low levels of albumin, rectal bleeding, and lesion progression to the left colon meant an increased probability of the moderate level. In addition, the decreased level of albumin had certain SHAP values in both predicting and excluding contribution, with some cases having higher SHAP values in predicting the moderate level. In the severe level prediction, albumin occupied the most important contribution of the model, followed by CRP/ALB, stool frequency, and hormone history. When compared to other levels’ predictions, previous use of hormones played a very important role in the discrimination of this category.

The UCEIS-based XGBoost prediction model was also analyzed by SHAP (Figure 5). The analysis revealed that the variable contributing most to the overall model was albumin, followed by CRP/ALB, rectal bleeding, and pulse rate, which are basically the same as the important indicators of MES based model. In contrast to MES, albumin primarily contributed to the discrimination of the moderate level, CRP/ALB contributed to that of the severe level, whereas rectal bleeding primarily contributed to that of the remission level, and pulse rate primarily played an important role in the remission and mild level. It is noteworthy that, except albumin, rectal bleeding, pulse rate, hematocrit, ESR, plateletcrit, and monocyte, the remaining variables contributed essentially nothing to the determination of the remission level. Further analysis of all variables SHAP values at each classification level showed that negative rectal bleeding and normal range of albumin had strong efficacy in predicting the remission level, while a low level of CRP/ALB had strong efficacy in predicting the mild level, and lower pulse rate had strong efficacy in excluding the mild level. At the mild level, the neutrophils ranked third in importance in the prediction model, and a high-level neutrophil contributed significantly to the prediction of a non-mild level. At the moderate level, the top four variables contributing to the model were albumin, disease duration, platelets, and stool frequency. Albumin showed a higher level of SHAP values in non-moderate levels. At the severe level, CRP/ALB occupied the most important variable again and showed strong efficacy in excluding the severe level. Albumin, decrease of weight, and ESR were followed by it, mainly showing that high-level albumin, a lower decrease of weight, and low-level ESR could exclude the severe level. Of note is that rectal bleeding played little role in predicting moderate and severe levels.

## 4 Discussion

Given the importance of long-term monitoring for UC patients, there are a dire need and challenges for developing better prediction tools to evaluate endoscopic disease activity in a non-invasive approach. In the present study, we used four ML algorithms to develop and validate non-invasive variables predictive models for UC patients. RF and XGBoost approach outperformed conventional LR models in predicting endoscopic disease activity, and they demonstrated favorable performance as an effective non-invasive tool for evaluating endoscopic disease activity. The model was developed from routinely collected clinical data and can be widely adopted and used, and has the advantage of predicting all groups simultaneously as one multi-label classifier. Moreover, with the expansion of the database, the model can be continuously improved and optimized for better precision and effectiveness. Such kind of efficient predictive models may be able to bring great convenience to disease management in patients with UC.

As already mentioned, endoscopic evaluation is often needed for monitoring disease recurrence and assessing the therapeutic effect in UC patients. This non-invasive predictive model is a meaningful tool for patients with UC, especially inactive patients. The management of inactive UC patients is primarily done in the outpatient setting, including part of self-management. The management of these patients becomes more difficult in the setting of the COVID-19 epidemic since most countries had reduced outpatient clinics and endoscopy (8). At the time of disease progression in patients during the remission period, symptoms may be infrequent and mild in character, colonoscopy is unvalued or even resisted by them. Nevertheless, the change in disease severity is directly correlated with clinical relapse and endoscopic exacerbation in patients with UC, which requires prompt therapeutic intervention (43, 44). Even though a large number of laboratory indicators are used in the model, compared to unconventional tests such as FC, the laboratory tests included in this model can be completed in basic hospitals or clinics. Therefore, the model in our study provides straightforward access for UC patients’ management, which can help patients to judge the endoscopic disease activity in time and effectively, and guide them to perform a timely colonoscopy. In addition, it also can contribute to the assessment of therapeutic effects in UC patients, and reduce the number of unnecessary invasive examinations.

Previous studies of evaluating endoscopic disease activity in UC have mainly focused on clinical scoring, biochemical measures, or building multi-index prediction models (10, 12, 45). However, the clinical scoring methods, such as the Seo Index and simple clinical colitis activity index correlate poorly with endoscopic disease activity (17). Currently, various biomarkers have been reported in this area, some of them were widely used in clinical practice, while others were limited to laboratory tests. The former includes FC, CRP, serum albumin to globulin ratio (AGR), and so on. FC and CRP have been widely studied and play a possible role in evaluating disease activity and monitoring medication response. Wang et al. proposed AGR as a marker for evaluating disease severity (46). However, their prediction value in UC was limited, they do not estimate the severity of UC accurately, nor are they sensitive/specific enough to monitor disease progression (13, 16, 47). The latter biomarkers include serum free thiols (R-SH), leucine-rich alpha-2 glycoprotein (LRG), IFN-γ, TNF-α, and other cytokines (10, 48, 49). Besides the sensitivity and specificity issue, another outstanding question is the challenge of generalization. Due to the COVID-19 epidemic and consumable costs, even FC is still not fully popularized currently, so it is more difficult to promote these biomarkers clinically. Similarly, the potential transcriptional blood biomarkers-based diagnosis still has a long way to go before it can be applied in the clinical setting (50). Compare with a single biomarker, most multi-index prediction models showed superior sensitivity, specificity, and accuracy. For example, studies by Bourgonje et al. (17), Langhorst et al. (51), and af Björkesten et al. (52) have shown that multi-parameter models outperform single-parameter. The majority of previous studies were applied to conventional methods, and the selected variables in those studies varied from clinical presentation to biochemical markers and imaging data. However, due to the limitations caused by the methodology and data collection inconsistencies, ideal linear regression or multiple regression models have restricted generalizability. In recent years, the promising results of ML applied in IBD have been obtained in many studies. The methods of model development, such as SVMs, decision trees, RF, gradient boosting, and neural network approach were applied in differential diagnosis, predicting prognosis, and therapeutic decisions of IBD (21). However, few studies have applied ML to evaluating endoscopic disease activity in patients with UC. In this study, we compared several ML methods and determine the optimal method for predicting patients’ endoscopic disease activity. The ML approach provides more accurate predictive power than conventional methods. Moreover, with the advantage of widely clinically applicable variables, ML algorithms can update themselves with the latest clinical data for higher accuracy, and achieve a more generalized non-linear model.

In this study, the amount of data for remission and mild endoscopic disease activity is relatively small, which may produce overfitting for the ML algorithm. The SMOTE method has been used for solving the imbalance problem in this study. During model training, data from the remission, mild, and severe UC groups were upsampled by using the SMOTE method, the data from each group reached an equal number and it improved the models’ AUC after sampling in our study. Data imbalance is a common problem during practical clinical studies, and most retrospective studies face this situation, which may influence the mining of the database for valid information. Though not exempt from intrinsic limitations, SMOTE can help solve the problem of dataset imbalance in the medical field as demonstrated by previous research, such as in the context of type 2 diabetes prediction (53), lung nodule recognition (54), and postoperative delayed remission prediction (55). In the present study, by comparing the model performance based on SMOTE data and the original data, we can find that the model performance has improved after SMOTE. It is interesting that some models show a decrease in accuracy after SMOTE but an increase in AUC. The reason for this may be that SMOTE improves the imbalance of the data and reduces the overfitting of the model, thus improving the AUC. And for ML models with unbalanced data, the improvement of AUC is more important than accuracy (56). Moreover, the ranking of the data after SMOTE in terms of parameter importance is consistent with the clinical practice, which also proves the feasibility of the SMOTE method.

Many emerged ML models are black-box models that lack variables relational analysis for clinical applications, and the model in our study suffers from this problem as well. Therefore, we introduced SHAP, an effective method for parametric interpretation of ML models, to explain the output prediction model, which provides a convincing interpretation of the relationships between non-linear variables (42). As an all-powerful approach to model interpretability, SHAP can work for both global and local interpretations. SHAP analysis of the model confirmed the importance of albumin, rectal bleeding, and CRP/ALB in evaluating the disease activity of UC, consistent with previous studies (10, 51). Further classification analysis revealed that different variables have their own roles in evaluating the active or remission of the disease. For example, under the MES, rectal bleeding and pulse rate, which were important in predicting remission level, were relatively ineffective in predicting severe level. Also, a similar situation is observed for rectal bleeding, pulse rate, and disease location under the UCEIS. This suggests that the change in endoscopic disease activity of UC is not adequately characterized by a single variable. This phenomenon can partly reflect the fact that the development of UC disease is not simply a linear change or a gradual accumulation of inflammation, but a complex and multi-factor intertwined result. It is relatively difficult to find a single variable to globally determine the endoscopic disease activity but requires a comprehensive and dynamic evaluation. The ML model we have chosen can partially mimic these complex relationships, making it possible to predict endoscopic disease activity through a single model. Besides this, after SHAP analysis, the clinicians can be guided to pay attention to the targeted variables when handling patients in remission or active phase, which is more conducive to the disease status evaluation. Moreover, the variables in our study are covered by many large cohort studies, the model can be better refined by incorporating data from previous experimental studies. Addressing the ethical and data issues involved will provide an opportunity for further research.

The present study employed both MES and UCEIS score systems in assessing the endoscopic disease activity of UC. Since it is not possible to make a correct objective assessment of the mucosa, different score systems have gradually been developed. MES and UCEIS are the two score systems that are widely developed in the clinic area currently (11, 57). MES is the most widely used endoscopic index due to its simplicity, and it has good inter-observer consistency (11). However, UCEIS is more advantageous for the subclassification of the patients in the active phase (58). Previous studies showed that UCEIS was better than MES for the subtle detection of mucosal changes, especially in predicting the rate of colectomy in patients with acute severe UC (58–60). In the present study, the results showed the model based on MES performed better than UCEIS-based models. This probably resulted from a more severe data imbalance in the UCEIS, which may lead to the overfitting of the model. Although we avoided overfitting the model by setting the relevant parameters and using SMOTE, the influence of the basic data on the model was still critical. Under both scoring systems, RF and XGboost models outperformed the conventional LR-based model and other ML algorithm models, indicating that RF and XGBoost are more suitable for predictive modeling of endoscopic disease activity in UC patients on the basis of clinical and laboratory tests. Then the variable importance analysis revealed that albumin, CRP/ALB, and rectal bleeding played important roles in both MES and UCEIS-based models. The SHAP method explained the model while reflecting the different details in the two scoring models. Comparing the SHAP contributions revealed the above three variables had a more balanced contribution to each classification under the MES than the UCEIS, which means UCEIS was relatively more sensitive to the distinction between different active phases, this might be the reason why UCEIS is more effective for subclassification. In addition, stool frequency, disease duration, urea, mean corpuscular volume, and history of 5-ASA had different importance in the MES and UCEIS. The causes for these differences were not well understood and need further investigation.

There are some limitations to the present study. One limitation of our study was the limited amount of data in some groups. Although the problem of class imbalance had been tackled by employing SMOTE in our study, a much larger population sample size would be needed to simulate the interactions among the variables. Meanwhile, this study was a cross-sectional study, the efficacy of the model for evaluating disease improvement after treatment cannot be totally reflected, future prospective studies to evaluate change in our machine-learning prediction models also correlate with changes in endoscopic inflammation after treatment, which hope to enlargement the dataset and reflect these changes with sensitivity and specificity. Second, as our study was a retrospective analysis, data on FC was missing. These laboratory indicators have been shown to be good predictive markers of disease severity (1, 61). If they could be included in further study, a more efficient model can be built in the future.

## 5 Conclusion

In conclusion, the use of the ML model containing multiple clinical and laboratory variables can serve as an effective non-invasive approach to predicting endoscopic disease activity for patients with long-standing UC, which can aid in determining individual treatment and follow-up strategies as well. For the first time, ML algorithms were introduced to UC endoscopic disease activity prediction, moreover, the application of RF, XGBoost, and SMOTE algorithms had a good performance on the modeling. An interactive platform based on these models can be further developed, patients will interact conveniently and can in turn help to improve the database at the same time. It also will spur the development of digital health in this field.

## Data availability statement

The raw data supporting the conclusions of this article will be made available by the authors, without undue reservation.

## Author contributions

XL: conceptualization, methodology, software, validation, formal analysis, data curation, and writing—original draft preparation. LY: methodology, validation, formal analysis, data curation, writing—review and editing, and visualization. XW: validation, data curation, and writing—review and editing. CO: formal analysis, investigation, and data curation. CW: conceptualization, formal analysis, investigation, writing—review and editing, and funding acquisition. JC: software, writing—original draft preparation, and visualization. JZ: conceptualization, resources, data curation, supervision, and project administration. GL: conceptualization, methodology, resources, supervision, and project administration. All authors have read and agreed to the published version of the manuscript.
