# Supplementary material for: Predictive models for endoscopic disease activity in patients with ulcerative colitis: Practical machine learning-based modeling and interpretation
Source: Front Med (Lausanne). 2022 Dec 21;9:1043412. doi: 10.3389/fmed.2022.1043412 (PMC9810755; doi:10.3389/fmed.2022.1043412)
Supplement: Supplementary file 1 [file Data_Sheet_1.pdf]

Supplementary Material

1 Supplementary

Supplementary Table.

Supplementary Table1: Ranking of variable importance based on Recursive Feature Elimination (RFE) feature reduction strategies.

Variables	Rank in MES	MES-SMOTE	Rank in UCEIS	UCEIS-SMOTE
Albumin	1	1	1	1
CRP/ALB	2	2	2	2
Urea	3		-	-
Disease duration	-	-	4	9
Hematocrit	4	5	14	10
CRP	5	8	6	4
ESR	6	4	5	3
Platelets	7	9	7	7
Monocyte	8	11	12	12
Pulse rate	9	6	11	5
White blood cells	10	16	8	13
Plateletcrit	11	14	13	14
Stool frequency	12	3	19	11
Hemoglobin	13	18	16	17
Neutrophils	14	12	3	8
Mean corpuscular volume	15	10	-	-
Lymphocyte	16	20	15	15
Weight	17	19	9	16
Rectal bleeding	18	7	17	6
Mean platelet volume	19	21	10	18
Decrease of weight	20	22	18	19
Hormone	21	17	21	22
Disease location	22	13	20	20
5-ASA	-	-	23	23
Immunomodulator therapy	23	23	22	21

ESR: erythrocyte sedimentation rate; CRP: C-reactive protein; ALB: Albumin; 5-ASA: 5-aminosalicylic acid.

Supplementary Table 2.

Supplementary Table 2: Optimization results of hyperparameters for each model.

Algorithms	Parameters	MES Original	MES SMOTE	UCEIS Original	UCEIS SMOTE
RF	Number of trees （n_estimators）	36	44	50	64
	Maximum tree depth (max_depth)	12	8	11	16
	Max features (max_features)	5	9	6	4
	Minimum number of samples required to split an internal leaf node (min_samples_split)	2	2	2	2
XGBoost	Number of trees (n_estimators)	52	26	36	88
	Learning rate (eta)	0.1	0.1	0.1	0.1
	Maximum tree depth (max_depth)	1	4	4	4
	Number of iterations （num_boost_round）	1	1	1	1
MLP	Hidden layers	1	1	1	1
	Number of nodes	46	40	54	38

RF: random forests; XGBoost, extreme gradient boost; MLP: multilayer perceptron; MES: mayo endoscopic subscore; UCEIS: ulcerative colitis endoscopic index of severity; SMOTE: synthetic minority oversampling technique.

Supplementary Figures.

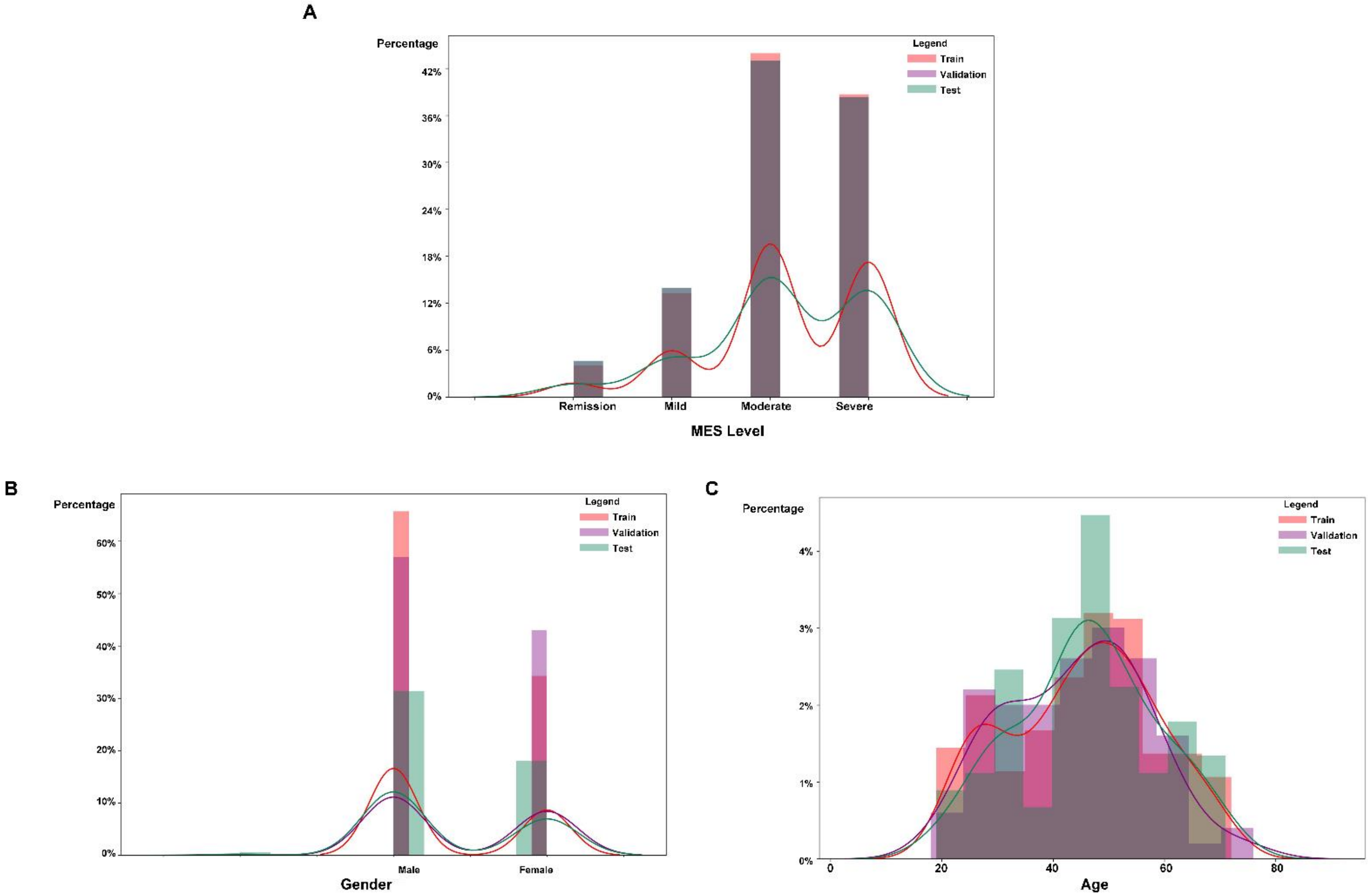

Supplementary Figure 1. Distribution of MES level (A), gender (B), and age (C) of patients in the training set, validation set, and test set in MES data. The area of the bar represents the percentage of this category in the total population, and the lines represent distribution curves. The distribution of patients in the training set, validation set, and test set are basically the same.

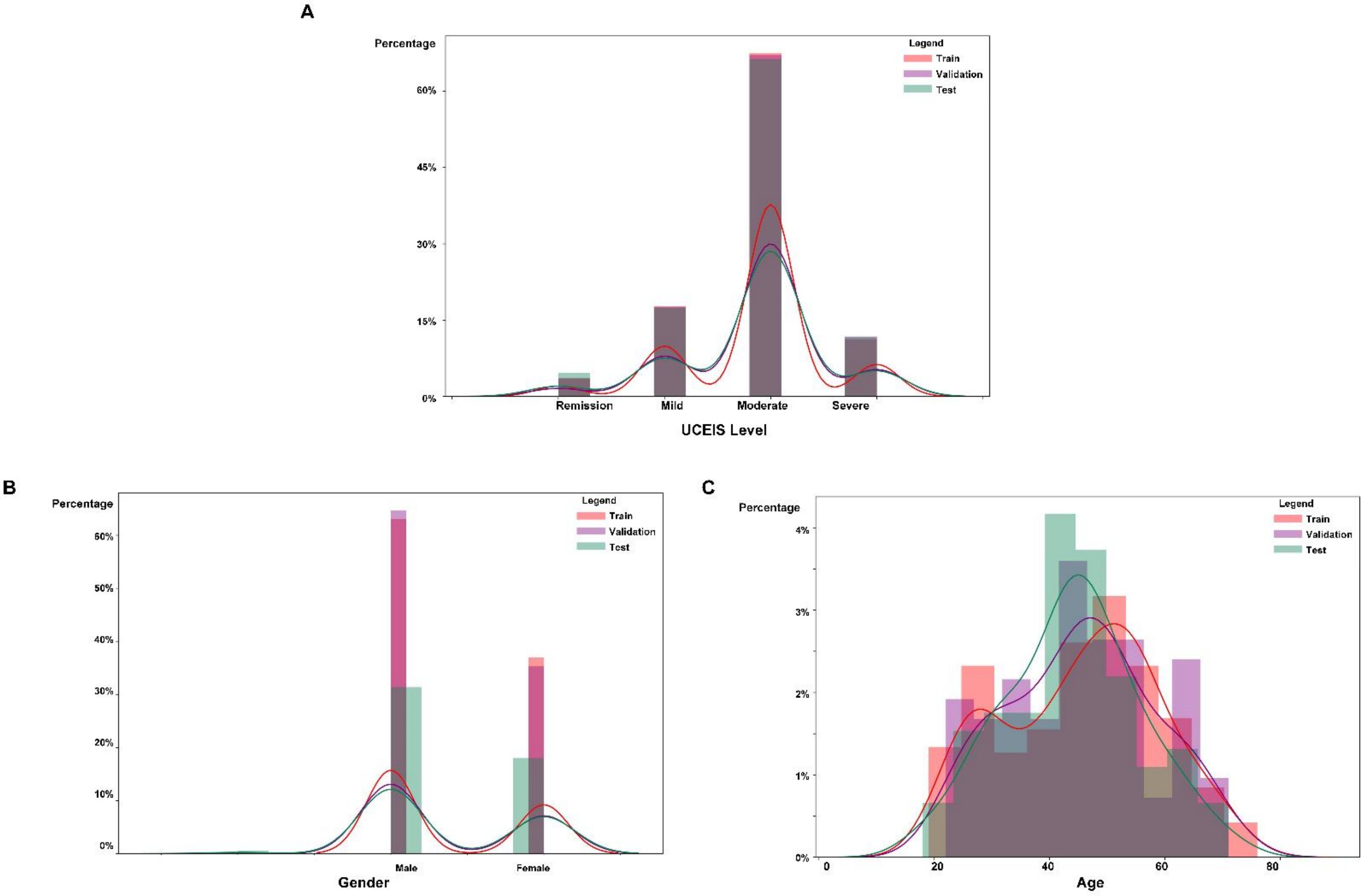

Supplementary Figure 2. Distribution of UCEIS level (A), gender (B), and age (C) of patients in the training set, validation set, and test set in UCEIS data. The area of the bar represents the percentage of this category in the total population, and the lines represent distribution curves. The distribution of patients in the training set, validation set, and test set are basically the same.
